# Supplementary material for: Dynamics of spike-specific neutralizing antibodies across five-year emerging SARS-CoV-2 variants of concern reveal conserved epitopes that protect against severe COVID-19
Source: Front Immunol. 2025 Feb 18;16:1503954. doi: 10.3389/fimmu.2025.1503954 (PMC11876060; doi:10.3389/fimmu.2025.1503954)
Supplement: Supplementary file 1 [file DataSheet1.pdf]

Supplemental Table. S1

Spike protein position	Start position in sequence	End position in sequence	Sequence
S_{13-37}	13	37	SQCVNLTTRTQLPPAYTNSFTRGVY
$S_{287-317}$	287	317	DAVDCALDPLSETKCTLKSFTVEKGIYQTSN
$S_{329-356}$	329	356	FPNITNLCPFGEVFNATRFASVYAWNRK
$S_{338-363}$	338	363	FGEVFNATRFASVYAWNRKRISNCVA
$S_{369-393}$	369	393	YNSASFSTFKCYGVSPTKLNDLCFT
$S_{440-470}$	440	470	NLDSKVGGNYNYLYRLFRKSNLKPFERDIST
$S_{456-485}$	456	485	FRKSNLKPFERDISTEIYQAGSTPCNGVEG
$S_{471-501}$	471	501	EIYQAGSTPCNGVEGFNCYFPLQSYGFQPTN
$S_{524-558}$	524	558	VCGPKKSTNLVKNKCVNFNFNGLTGTGVLTESNKK
$S_{544-578}$	544	578	NGLTGTGVLTESNKKFLPFQQFGRDIADTTDAVRD
$S_{565-598}$	565	598	QFGRDIADTTDAVRDPQTLEILDITPCSFGGVSVI
$S_{601-628}$	601	628	GTNTSNQVAVLYQDVNCTEVPVAIHADQ
$S_{614-640}$	613	640	QDVNCTEVPVAIHADQLTPTWRVYSTGS
$S_{802-819}$	802	819	FSQILPDPSKPSKRSFIE
$S_{888-909}$	888	909	FGAGAALQIPFAMQMAYRFNGI
$S_{1133-1160}$	1133	1160	VNNTVYDPLQPELDSFKEELDKYFKNHT
$S_{1145-1172}$	1145	1172	LDSFKEELDKYFKNHTSPDVDLGDISGI

<u>Supplemental Table S1</u>. The Amino acid sequences and selection criteria of B-cell epitopes from the spike protein of SARS-CoV-2. The table presents the amino acid sequences of 17 B-cell epitopes derived from the spike protein of SARS-CoV-2. These epitopes were selected based on their high conservation across various coronaviruses, including SARS-CoV-2, the four major "common cold" coronaviruses (CoV-OC43, CoV-229E, CoV-HKU1, and CoV-NL63), and SARS-like coronaviruses (SL-CoVs) isolated from bats, civet cats, pangolins, and camels. Additionally, epitope selection considered the likelihood of each linear epitope being exposed on the surface of infected target cells.

Supplemental Fig. S1

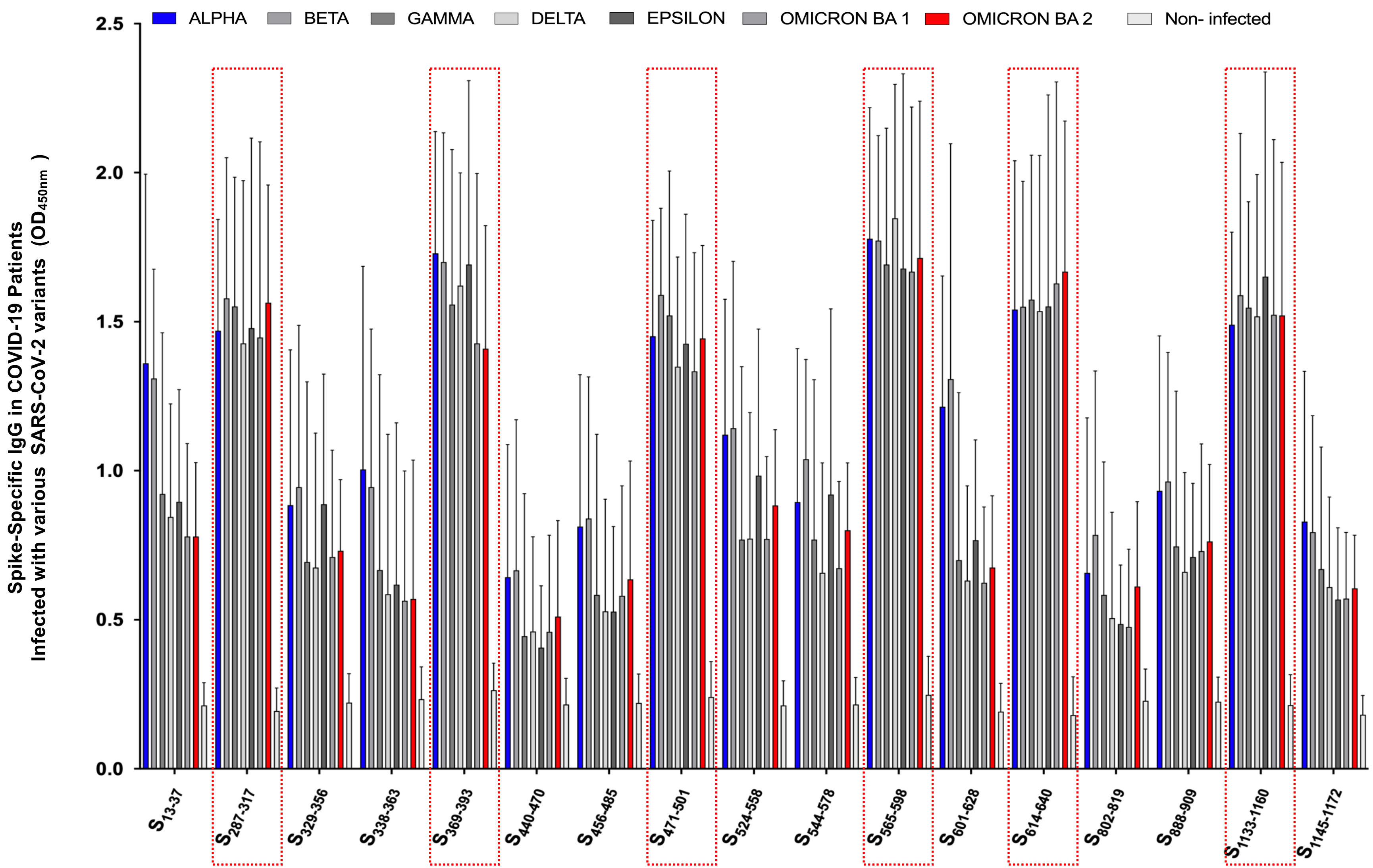

Supplemental Figure S1: Evaluation of IgG binding to conserved B cell "asymptomatic" epitopes. Graph shows the optical density for anti SARS-CoV-2 peptide specific IgG measured in sera from different groups of COVID-19 infected with highly pathogenic SARS-CoV-2 variants of concern. Dotted lines indicate selected peptides demonstrating notably high immunogenicity among the 17 immunodominant B cell peptides analyzed.

Supplemental Fig. S2

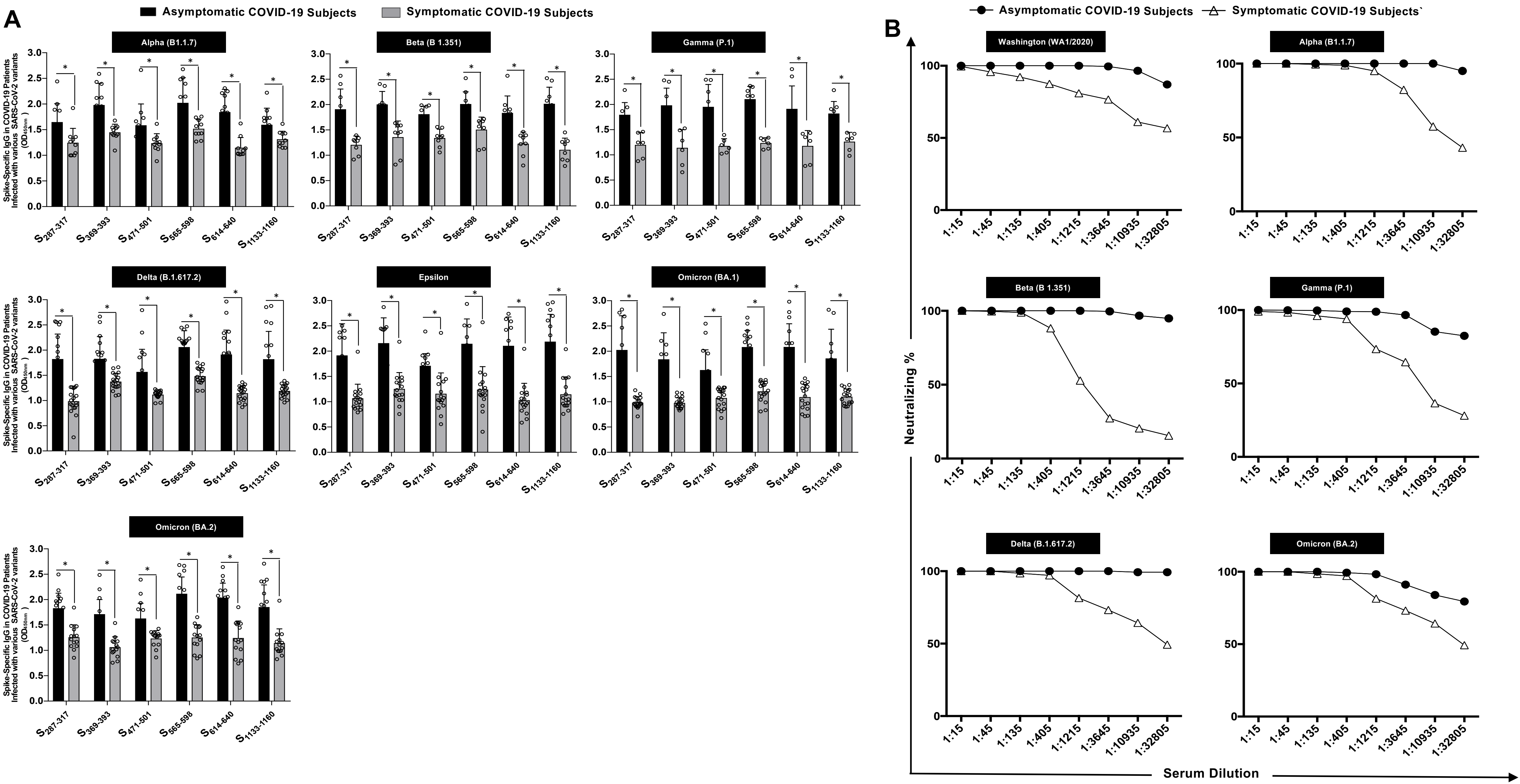

Supplemental Figure S2. Severity-dependent immune responses against 'universal' B-cell epitopes in COVID-19 patients exposed to different SARS-CoV-2 variants of concern: Highly conserved COVID-19 peptide immunogenicity evaluation. Bar graphs show the peptide binding IgG level for the 6 “universal” B cell epitopes meseaured by ELISA as (*panel* **A**). Serum samples were obtained from COVID-19 patients infected with various SARS-CoV-2 variants of concern (VOC) including Alpha (B.1.1.7), Beta (B.1.351), Epsilon (B.1.427/B.1.429), Delta (B.1.617.2), and Omicron (BA.1 and BA.2), and segregated into two distinct groups based on severity level, categorized as "Asymptomatic" and "Symptomatic". Neutralization (%) by sera from these patients against the different VOCs of SARS-CoV-2: Alpha (B.1.1.7), Beta (B.1.351), Epsilon (B.1.427/B.1.429), Delta (B.1.617.2), and Omicron (BA.2) is presented in (*panel* **B**)**.** Bars represent means ± SEM. Data were analyzed by student's *t*-test and multiple t-tests. Results were considered statistically significant at $P < 0.05$. Statistical correction for multiple comparisons was applied using the Holm-Sidak method.

Supplemental Fig. S3

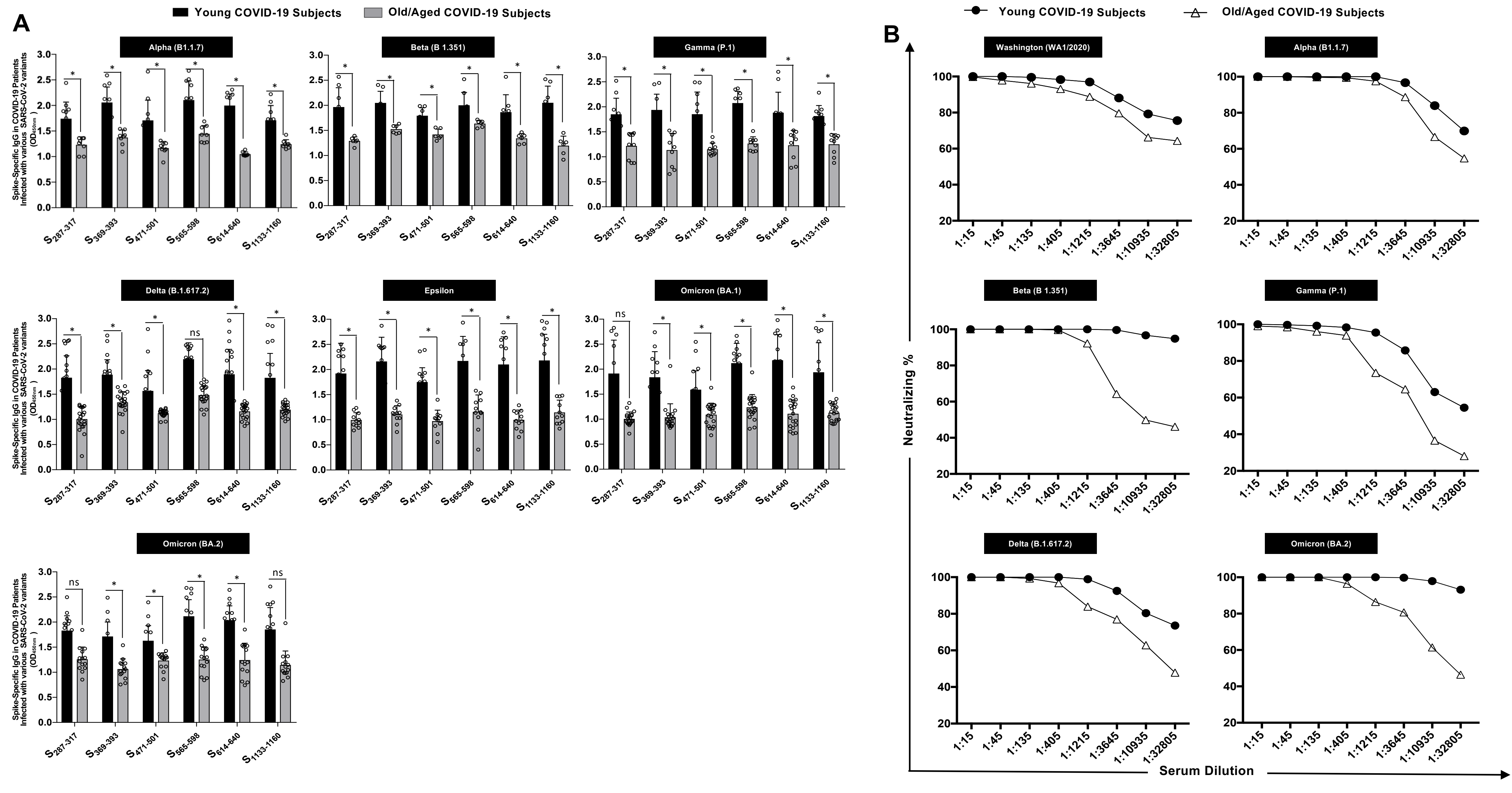

<u>Supplemental Figure S3</u>. Age-dependent immune responses against 'universal' B-cell epitopes in COVID-19 patients exposed to different SARS-CoV-2 variants of concern: Highly conserved COVID-19 peptide immunogenicity evaluation. Bar graphs represent the peptide binding IgG level for the 6 “universal” B cell epitopes meseaured by ELISA as shown in *panel* (**A**). Serum was obtained from COVID-19 patients that were infected with one of the following six SARS-CoV-2 different variants of concern (VOC) Alpha (B.1.1.7), Beta (B.1.351), Epsilon (B.1.427/B.1.429), Delta (B.1.617.2), and Omicron (BA.1) and Omicron (BA.2) and then segregated into two distinct age groups, categorized as "Old" and "Young". The (**B**) *panel* represents Neutralization (%) by sera from COVID-19 patients that were infected with one of the six different variants of concern (VOC) of SARS-CoV-2 and then segregated into two distinct age groups; "Old" and "Young" against Alpha (B.1.1.7), Beta (B.1.351), Epsilon (B.1.427/B.1.429), Delta (B.1.617.2), and Omicron (BA.2)**.** Bars represent means ± SEM. Data were analyzed by student's *t*-test and multiple t-tests. Results were considered statistically significant at $P < 0.05$. Statistical correction for multiple comparisons was applied using the Holm-Sidak method.

Supplemental Fig. S4

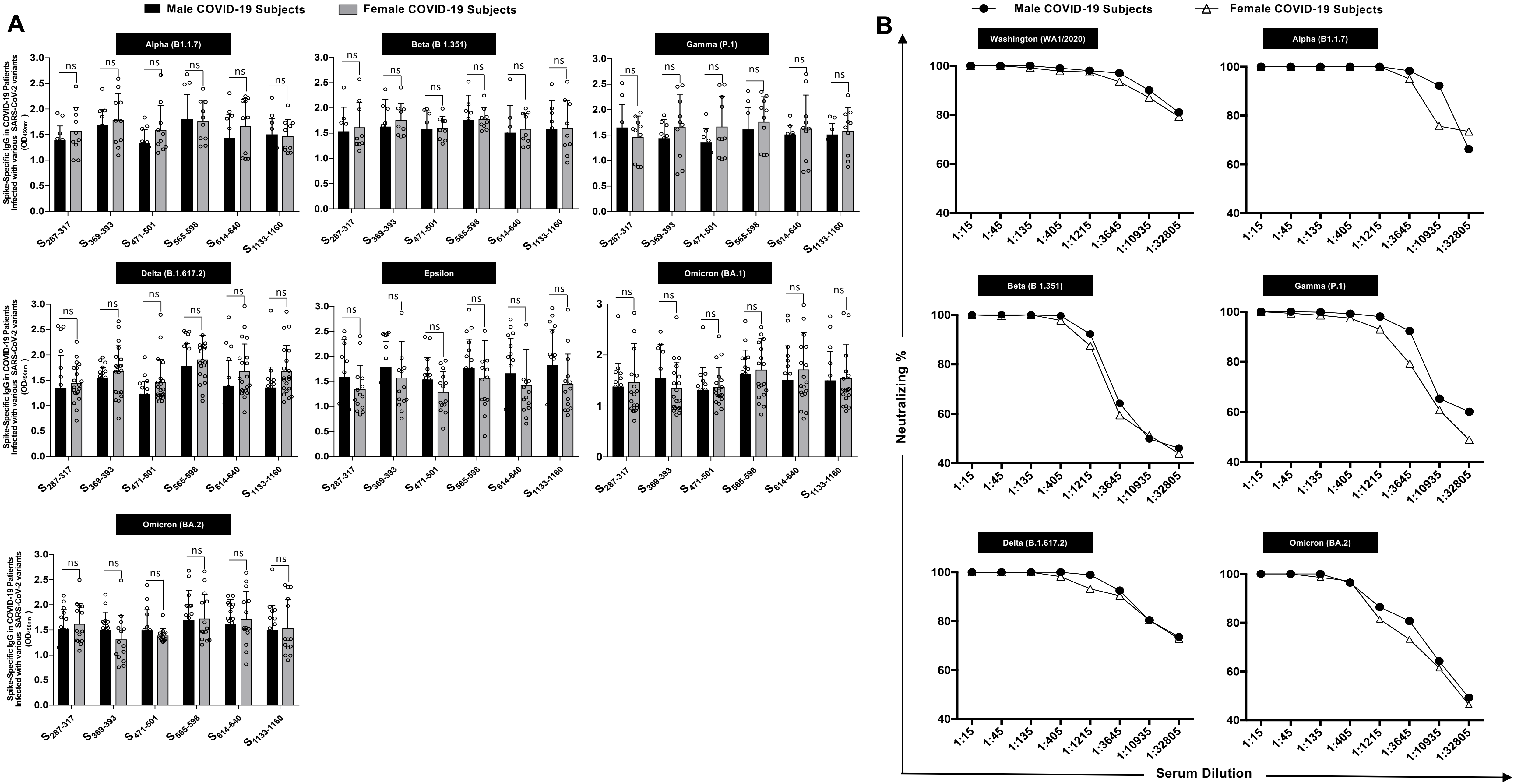

Supplemental Figure S4. Gender-dependent immune responses against 'universal' B-cell epitopes in COVID-19 patients exposed to different SARS-CoV-2 variants of concern: Highly conserved COVID-19 peptide immunogenicity evaluation. Similar to (**Fig. 2**) and (**Fig. 3**), bar graphs illustrate the peptide binding IgG level for the 6 "universal" B cell epitopes measured by ELISA (*panel* **A**). Serum samples were collected from COVID-19 patients infected with various SARS-CoV-2 variants of concern (VOC) and categorized into two distinct groups based on gender. The (**B**) *panel* shows neutralization (%) by sera from these patients against the different SARS-CoV-2 VOCs Alpha (B.1.1.7), Beta (B.1.351), Epsilon (B.1.427/B.1.429), Delta (B.1.617.2), and Omicron (BA.2). Bars represent means ± SEM. Data were analyzed using student's t-test and multiple t-tests. Results were considered statistically significant at $P < 0.05$, with statistical correction applied using the Holm-Sidak method.

Supplemental Fig. S5

$S_{287\text{-}317}$

h-CoV-2/Wuhan (MN908947.3)	D	A	V	D	C	A	L	D	P	L	S	E	T	K	C	T	L	K	S	F	T	V	E	K	G	I	Y	Q	T	S	N
h-CoV-2/WA/USA2020 (OQ294668.1)	D	A	V	D	C	A	L	D	P	L	S	E	T	K	C	T	L	K	S	F	T	V	E	K	G	I	Y	Q	T	S	N
h-CoV-2/Alpha (B1.1.7) (OL689430.1)	D	A	V	D	C	A	L	D	P	L	S	E	T	K	C	T	L	K	S	F	T	V	E	K	G	I	Y	Q	T	S	N
h-CoV-2/Beta (B 1.351) (MZ314998)	D	A	V	D	C	A	L	D	P	L	S	E	T	K	C	T	L	K	S	F	T	V	E	K	G	I	Y	Q	T	S	N
h-CoV-2/Gamma (P.1) (MZ427312.1)	D	A	V	D	C	A	L	D	P	L	S	E	T	K	C	T	L	K	S	F	T	V	E	K	G	I	Y	Q	T	S	N
h-CoV-2/Delta (B.1.617.2) (OK091006.1)	D	A	V	D	C	A	L	D	P	L	S	E	T	K	C	T	L	K	S	F	T	V	E	K	G	I	Y	Q	T	S	N
h-CoV-2/Omicron (B.1.1.529) (OM570283.1)	D	A	V	D	C	A	L	D	P	L	S	E	T	K	C	T	L	K	S	F	T	V	E	K	G	I	Y	Q	T	S	N
h-CoV-2/Omicron (BA.2) (OM617939.1)	D	A	V	D	C	A	L	D	P	L	S	E	T	K	C	T	L	K	S	F	T	V	E	K	G	I	Y	Q	T	S	N

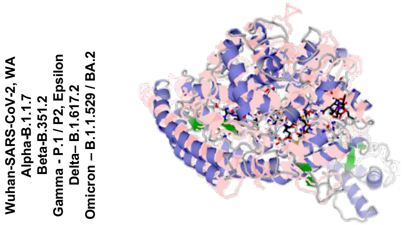

Interaction similarity = 29

$S_{369\text{-}393}$

h-CoV-2/Wuhan (MN908947.3)	Y	N	S	A	S	F	S	T	F	K	C	Y	G	V	S	P	T	K	L	N	D	L	C	F	T
h-CoV-2/WA/USA2020 (OQ294668.1)	Y	N	F	A	P	F	F	A	F	K	C	Y	G	V	S	P	T	K	L	N	D	L	C	F	T
h-CoV-2/Alpha (B1.1.7) (OL689430.1)	Y	N	S	A	S	F	S	T	F	K	C	Y	G	V	S	P	T	K	L	N	D	L	C	F	T
h-CoV-2/Beta (B 1.351) (MZ314998)	Y	N	S	A	S	F	S	T	F	K	C	Y	G	V	S	P	T	K	L	N	D	L	C	F	T
h-CoV-2/Gamma (P.1) (MZ427312.1)	Y	N	S	A	S	F	S	T	F	K	C	Y	G	V	S	P	T	K	L	N	D	L	C	F	T
h-CoV-2/Delta (B.1.617.2) (OK091006.1)	Y	N	S	A	S	F	S	T	F	K	C	Y	G	V	S	P	T	K	L	N	D	L	C	F	T
h-CoV-2/Omicron (B.1.1.529) (OM570283.1)	Y	N	L	A	P	F	F	A	F	K	C	Y	G	V	S	P	T	K	L	N	D	L	C	F	T
h-CoV-2/Omicron (BA.2) (OM617939.1)	Y	N	F	A	P	F	F	A	F	K	C	Y	G	V	S	P	T	K	L	N	D	L	C	F	T

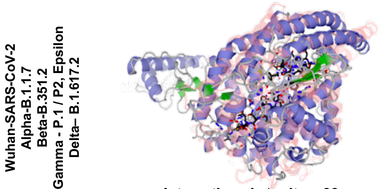

Interaction similarity = 30

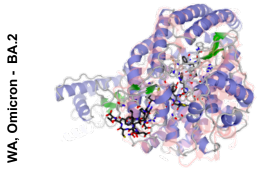

Interaction similarity = 33

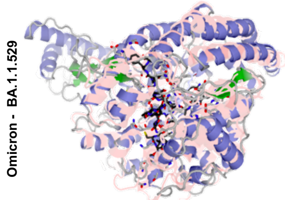

Interaction similarity = 34

$S_{471\text{-}501}$

h-CoV-2/Wuhan (MN908947.3)	E	I	Y	Q	A	G	S	T	P	C	N	G	V	E	G	F	N	C	Y	F	P	L	Q	S	Y	G	F	Q	P	T	N
h-CoV-2/WA/USA2020 (OQ294668.1)	E	I	Y	Q	A	G	N	K	P	C	N	G	V	A	G	V	N	C	Y	F	P	L	Q	S	Y	G	F	R	P	T	Y
h-CoV-2/Alpha (B1.1.7) (OL689430.1)	E	I	Y	Q	A	G	S	T	P	C	N	G	V	E	G	F	N	C	Y	F	P	L	Q	S	Y	G	F	Q	P	T	Y
h-CoV-2/Beta (B 1.351) (MZ314998)	E	I	Y	Q	A	G	S	T	P	C	N	G	V	K	G	F	N	C	Y	F	P	L	Q	S	Y	G	F	Q	P	T	Y
h-CoV-2/Gamma (P.1) (MZ427312.1)	E	I	Y	Q	A	G	S	T	P	C	N	G	V	K	G	F	N	C	Y	F	P	L	Q	S	Y	G	F	Q	P	T	Y
h-CoV-2/Delta (B.1.617.2) (OK091006.1)	E	I	Y	Q	A	G	S	K	P	C	N	G	V	E	G	F	N	C	Y	F	P	L	Q	S	Y	G	F	Q	P	T	N
h-CoV-2/Omicron (B.1.1.529) (OM570283.1)	E	I	Y	Q	A	G	N	K	P	C	N	G	V	A	G	F	N	C	Y	F	P	L	R	S	Y	S	F	R	P	T	Y
h-CoV-2/Omicron (BA.2) (OM617939.1)	E	I	Y	Q	A	G	N	K	P	C	N	G	V	A	G	S	N	C	Y	S	P	L	R	S	Y	G	F	R	P	T	Y

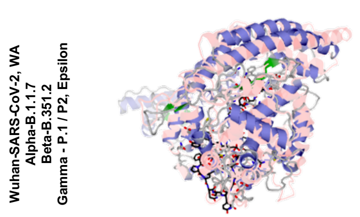

Interaction similarity = 31

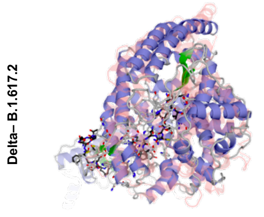

Interaction similarity = 30

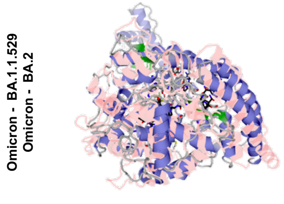

Interaction similarity = 31

$S_{565\text{-}598}$

h-CoV-2/Wuhan (MN908947.3)	Q	F	G	R	D	I	A	D	T	T	D	A	V	R	D	P	Q	T	L	E	I	L	D	I	T	P	C	S	F	G	G	V	S	V	I
h-CoV-2/WA/USA2020 (OQ294668.1)	Q	F	G	R	D	I	A	D	T	T	D	A	V	R	D	P	Q	T	L	E	I	L	D	I	T	P	C	S	F	G	G	V	S	V	I
h-CoV-2/Alpha (B1.1.7) (OL689430.1)	Q	F	G	R	D	I	D	D	T	T	D	A	V	R	D	P	Q	T	L	E	I	L	D	I	T	P	C	S	F	G	G	V	S	V	I
h-CoV-2/Beta (B 1.351) (MZ314998)	Q	F	G	R	D	I	A	D	T	T	D	A	V	R	D	P	Q	T	L	E	I	L	D	I	T	P	C	S	F	G	G	V	S	V	I
h-CoV-2/Gamma (P.1) (MZ427312.1)	Q	F	G	R	D	I	A	D	T	T	D	A	V	R	D	P	Q	T	L	E	I	L	D	I	T	P	C	S	F	G	G	V	S	V	I
h-CoV-2/Delta (B.1.617.2) (OK091006.1)	Q	F	G	R	D	I	A	D	T	T	D	A	V	R	D	P	Q	T	L	E	I	L	D	I	T	P	C	S	F	G	G	V	S	V	I
h-CoV-2/Omicron (B.1.1.529) (OM570283.1)	Q	F	G	R	D	I	A	D	T	T	D	A	V	R	D	P	Q	T	L	E	I	L	D	I	T	P	C	S	F	G	G	V	S	V	I
h-CoV-2/Omicron (BA.2) (OM617939.1)	Q	F	G	R	D	I	A	D	T	T	D	A	V	R	D	P	Q	T	L	E	I	L	D	I	T	P	C	S	F	G	G	V	S	V	I

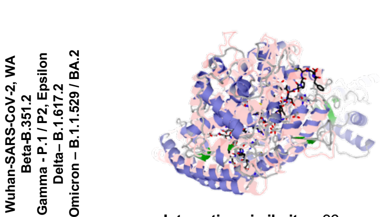

Interaction similarity = 36

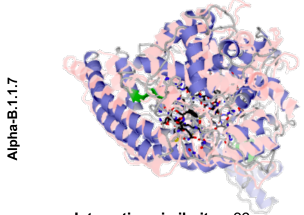

Interaction similarity = 36

$S_{614\text{-}640}$

h-CoV-2/Wuhan (MN908947.3)	Q	D	V	N	C	T	E	V	P	V	A	I	H	A	D	Q	L	T	P	T	W	R	V	Y	S	T	G	S
h-CoV-2/WA/USA2020 (OQ294668.1)	Q	G	V	N	C	T	E	V	P	V	A	I	H	A	D	Q	L	T	P	T	W	R	V	Y	S	T	G	S
h-CoV-2/Alpha (B1.1.7) (OL689430.1)	Q	G	V	N	C	T	E	V	P	V	A	I	H	A	D	Q	L	T	P	T	W	R	V	Y	S	T	G	S
h-CoV-2/Beta (B 1.351) (MZ314998)	Q	G	V	N	C	T	E	V	P	V	A	I	H	A	D	Q	L	T	P	T	W	R	V	Y	S	T	G	S
h-CoV-2/Gamma (P.1) (MZ427312.1)	Q	G	V	N	C	T	E	V	P	V	A	I	H	A	D	Q	L	T	P	T	W	R	V	Y	S	T	G	S
h-CoV-2/Delta (B.1.617.2) (OK091006.1)	Q	G	V	N	C	T	E	V	P	V	A	I	H	A	D	Q	L	T	P	T	W	R	V	Y	S	T	G	S
h-CoV-2/Omicron (B.1.1.529) (OM570283.1)	Q	G	V	N	C	T	E	V	P	V	A	I	H	A	D	Q	L	T	P	T	W	R	V	Y	S	T	G	S
h-CoV-2/Omicron (BA.2) (OM617939.1)	Q	G	V	N	C	T	E	V	P	V	A	I	H	A	D	Q	L	T	P	T	W	R	V	Y	S	T	G	S

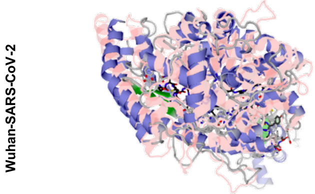

Interaction similarity = 35

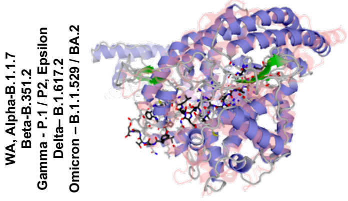

Interaction similarity = 35

$S_{1133\text{-}1160}$

h-CoV-2/Wuhan (MN908947.3)	V	N	N	T	V	Y	D	P	L	Q	P	E	L	D	S	F	K	E	E	L	D	K	Y	F	K	N	H	T
h-CoV-2/WA/USA2020 (OQ294668.1)	V	N	N	T	V	Y	D	P	L	Q	P	E	L	D	S	F	K	E	E	L	D	K	Y	F	K	N	H	T
h-CoV-2/Alpha (B1.1.7) (OL689430.1)	V	N	N	T	V	Y	D	P	L	Q	P	E	L	D	S	F	K	E	E	L	D	K	Y	F	K	N	H	T
h-CoV-2/Beta (B 1.351) (MZ314998)	V	N	N	T	V	Y	D	P	L	Q	P	E	L	D	S	F	K	E	E	L	D	K	Y	F	K	N	H	T
h-CoV-2/Gamma (P.1) (MZ427312.1)	V	N	N	T	V	Y	D	P	L	Q	P	E	L	D	S	F	K	E	E	L	D	K	Y	F	K	N	H	T
h-CoV-2/Delta (B.1.617.2) (OK091006.1)	V	N	N	T	V	Y	D	P	L	Q	P	E	L	D	S	F	K	E	E	L	D	K	Y	F	K	N	H	T
h-CoV-2/Omicron (B.1.1.529) (OM570283.1)	V	N	N	T	V	Y	D	P	L	Q	P	E	L	D	S	F	K	E	E	L	D	K	Y	F	K	N	H	T
h-CoV-2/Omicron (BA.2) (OM617939.1)	V	N	N	T	V	Y	D	P	L	Q	P	E	L	D	S	F	K	E	E	L	D	K	Y	F	K	N	H	T

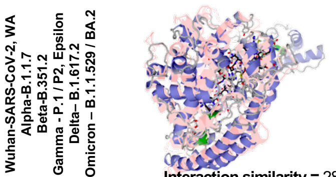

Interaction similarity = 28

<u>Supplemental Figure S5</u>. Docking of SARS-CoV-2 Spike glycoprotein-derived B cell epitopes to human ACE2 receptor: Molecular docking of 6 B-cell epitopes was performed, that were found to be highly immunodominant across all the known SARS-CoV-2 variants of concern. The B-cell epitopes were identified from the SARS-CoV-2 Spike glycoprotein, with ACE2 receptors. B cell epitopes are shown in ball and stick structures whereas the ACE2 receptor protein is shown as a template. $S_{471-501}$ and $S_{369-393}$ peptide epitopes possess receptor binding domain region specific amino acid residues. The prediction accuracy is estimated from a linear model as the relationship between the fraction of correctly predicted binding site residues and the template-target similarity measured by the protein structure similarity score and interaction similarity score (S_{Inter}) obtained by linear regression. S_{Inter} shows the similarity of amino acids of

the B-cell peptides aligned to the contacting residues in the amino acids of the ACE2 template structure. Higher S_{Inter} score represents a more significant binding affinity among the ACE2 molecule and B-cell peptides.
